# Duration of Hospitalization is Associated with the Gut Microbiome in Patients Undergoing Hematopoietic Stem Cell Transplantation: Early Results from a Randomized Trial of Home Versus Hospital Transplantation

**DOI:** 10.21926/obm.transplant.2503255

**Published:** 2025-08-25

**Authors:** Tessa M. Andermann, Ke Zeng, Sayal Guirales-Medrano, Adam Groth, Bhanu C. Ramachandran, Shan Sun, Alicia A. Sorgen, Lauren Hill, Amy T. Bush, Hongwei Liu, Corbin Jones, Jeffrey Roach, Brian P. Conlon, Gauri Rao, Nelson J. Chao, Anthony A. Fodor, Anthony D. Sung

**Affiliations:** 1 Division of Infectious Diseases, Department of Medicine, University of North Carolina at Chapel Hill, North Carolina, USA; 2 Department of Bioinformatics and Genomics, University of North Carolina at Charlotte Charlotte, North Carolina, USA; 3 Bioinformatics and Analytics Research Collaborative, School of Medicine, University of North Carolina at Chapel Hill, North Carolina, USA; 4 Division of Hematologic Malignancies and Cellular Therapy, Department of Medicine, Duke University Medical Center, North Carolina, USA; 5 Department of Biology, University of North Carolina at Chapel Hill, North Carolina, USA; 6 Center for Gastrointestinal Biology and Disease (CGIBD), Division of Gastroenterology and Hepatology, Department of Medicine, University of North Carolina, North Carolina, USA; 7 Department of Microbiology and Immunology, University of North Carolina at Chapel Hill, North Carolina, USA; 8 Department of Clinical Pharmacy, University of Southern California, California, USA; 9 Department of Medicine, Division of Hematologic Malignancies and Cellular Therapeutics, University of Kansas, Kansas, USA

**Keywords:** Microbiome, hematopoietic stem cell transplantation, blood and marrow transplantation

## Abstract

Home-based hematopoietic stem cell transplantation (HCT) is an innovative care model with growing interest, but its impact on the gut microbiome remains unexplored in a randomized setting. We present interim results from the first randomized controlled trials (RCT) evaluating the effect of HCT location—home versus hospital—on gut microbial diversity and antimicrobial resistance (AMR) gene carriage. We hypothesize that patients randomized to undergo home HCT would have higher gut taxonomic diversity and lower AMR gene abundance compared to those undergoing standard hospital HCT. We analyzed stool samples from the first 28 patients enrolled in ongoing Phase II RCTs comparing home (n = 16) and hospital (n = 12) HCT at Duke University using shotgun metagenomic sequencing to compare taxa and AMR gene composition between groups. We also performed a secondary analysis comparing patients who received transplants at outpatient infusion clinics versus inpatient standard HCT to evaluate the influence of hospitalization duration. In the primary RCT analysis, taxonomic and AMR gene α- and β-diversity were comparable between home and hospital groups, reflecting similar durations of hospitalization despite group allocation. In contrast, secondary analyses demonstrated that patients transplanted in outpatient infusion clinics who experienced significantly reduced hospitalization had higher gut taxonomic α-diversity and differential β-diversity, although AMR gene diversity remained unchanged. In summary, randomization by transplant location did not impact the gut microbiota to the same extent as the duration of hospitalization, although secondary analyses were heavily confounded. Even when taxonomic differences were observed, AMR genes were similar between groups. This RCT represents a novel investigation into how care setting influences the gut microbiome during HCT. Our findings suggest that hospital duration, rather than randomization allocation alone, is the primary driver of microbial disruption. These results underscore the potential for reducing hospital duration to mitigate microbiome injury, thereby informing future interventions to reduce infection risk and improve patient outcomes.

## 1. Introduction

Hematopoietic stem cell transplantation (HCT) is a potentially life-saving process that remains enormously burdensome for more than 22,000 individuals in the United States each year as of 2021 [1]. Hospital stays for HCT are often several weeks to months in duration and result in significantly decreased quality of life [2–5]. Patients in the hospital are also at increased risk for exposure to multidrug-resistant nosocomial pathogens with each day spent as inpatient, through contact with healthcare personnel and the hospital environment.

In contrast to hospital HCT, home HCT, delivers all post-HCT care in the home setting, offering patients comfort through a familiar environment [2–5]. Patients in home HCT protocols generally receive pre-HCT conditioning and stem cell infusion in the hospital or day hospital, followed by immediate discharge home. Daily visits to the patient home are performed by the healthcare team allowing for labs, vitals checks, and administration of medications. Prior case-controlled studies of home HCT in Sweden, for example, demonstrated lower rates of acute graft-versus-host disease (aGVHD), improved survival, and decreased medical costs [3, 4, 6, 7]. The transplant group at Duke University demonstrated in a small Phase I observational study that, while clinical outcomes (*e.g.*, aGVHD, infection) did not differ between trial patients undergoing HCT at home versus hospital controls, pre-HCT quality of life was preserved for home HCT recipients [5]. Phase II RCTs are ongoing by this same group (NCT02218151, NCT03667599) with the goal of comparing both clinical (aGVHD, infection) outcomes and gut microbial diversity between groups.

Changes in the diversity and taxonomic composition of the gut microbiome in patients undergoing HCT have long been implicated in the development of aGVHD, infection risk, and mortality among other important clinical outcomes [8]. Preserving gut microbial diversity may therefore be a modifiable factor with the potential to improve post-transplant outcomes and reduce complications such as infection and transplant-related mortality. Given that transplantation at home may reduce the risk of aGVHD, we hypothesized that home HCT patients would have less disruption to their gut microbiomes over time, demonstrating greater gut microbial diversity relative to hospital HCT controls. Herein we demonstrate the earliest results from the first randomized trial of patients undergoing HCT at home versus in the hospital, comparing changes in the gut microbiome over time. Knowing that the gut microbiome also serves as an important reservoir for multidrug-resistant organisms often acquired during hospitalization, we also asked whether patients undergoing HCT at home would demonstrate a decreased burden of antimicrobial resistance (AMR) genes in the organisms found in their gut as well.

## 2. Materials and Methods

### 2.1 Patient Cohort Selection

Samples for the study were obtained retrospectively from the first patients consented and enrolled in the Phase II trials of home versus hospital HCT at Duke between 1/1/2017 and 12/31/2020 [5]. Stools in the biorepository had previously been collected at pre-defined time points (pre-HCT, and days 0, 7, 14, 21, 28, 35, 60, 100, 180, 270, 365, and 730 after HCT). Due to small sample sizes, D28 and D35 samples were merged into a single bin labeled D30, referred to as “day +30”, used in the majority of the analyses.

All allogeneic and autologous patients in both home and hospital HCT cohorts received standard antimicrobial prophylaxis with a fluoroquinolone--either ciprofloxacin or levofloxacin--along with acyclovir, fluconazole, and trimethoprim-sulfamethoxazole as previously described [5].

Clinical metadata were collected prospectively from the electronic medical record and included date of transplantation, type of pre-HCT conditioning, donor type, graft source, duration of hospitalization, antimicrobial use, and date of aGVHD with aGVHD grade defined according to the IBMTR grading system [9]. Data for the study were last accessed for the study 30/11/2022. Authors did not have access to data that could identify individual participants during or after data collection.

### 2.2 Ethics Statement

Patient consent was required for inclusion in the studies and prior to collection of samples for research. Research activities were supported by the Institutional Review Boards of Duke University (IRB protocols #00051024, and #00089697) and the University of North Carolina at Chapel Hill (IRB protocol #20–2371).

### 2.3 Defining Transplant Location: Inpatient Versus Outpatient HCT

In the Duke clinical trials, both autologous and allogeneic HCT recipients were randomized to either home or hospital HCT Patients randomized to either home or hospital HCT received their stem cell infusions either in the hospital (“inpatient”) or in the infusion clinic (“outpatient”) as decided upon by their transplant physician. We determined that inpatient and outpatient groups almost perfectly differentiated patients who were hospitalized for longer (inpatient) or shorter (outpatient) durations. For the purposes of investigating the impact of duration of hospitalization on the microbiome and resistome, we compared these two groups as part of our secondary analysis.

### 2.4 Stool Sample Processing and Sequencing

#### 2.4.1 DNA Extraction

Stools were collected from patients and placed on ice, stored at 4°C for less than 24 hours before aliquoting into cryovials, and frozen at −80°C before DNA extraction. DNA isolation was performed using an optimized version of the QIAamp Fast DNA Stool Mini Kit protocol supplemented with 60 mg/mL lysozyme (Thermo Fisher Scientific) [10]. At the time of DNA extraction, samples were transferred to a 2 ml tube containing 200 mg of 106/500 μm glass beads (Sigma) and 0.5 ml of Qiagen PM_1_ buffer. Mechanical lysis was performed for 40 minutes on a Digital Vortex Mixer. After a 5 minutes centrifugation, 0.45 ml of supernatants was aspirated and transferred to a new tube containing 0.15 ml of Qiagen IRS solution. The suspension was incubated at 4°C overnight. After a brief centrifugation, supernatant was aspirated and transferred to deep well plates containing 0.45 ml of Qiagen binding buffer supplemented with Qiagen ClearMag Beads. DNA was purified using the automated KingFisher^™^ Flex Purification System and eluted in DNase free water. ZymoBIOMICS Gut Microbiome Standard was used as the positive control.

#### 2.4.2 Library Preparation and Metagenomic Sequencing

DNA isolated from samples was assessed for sample quality and DNA fragment size on a TapeStation (Agilent). DNA libraries for sequencing were prepared using the Illumina DNA Seq protocol (formerly, Nextera Flex). Samples were pooled in groups of 96 and then checked on a MiSeq Nano (Illumina Inc.) for pool balance. The pool was then rebalanced and sequenced as 150 paired-end reads on a NovaSeq 6000 S4 flowcell (Illumina Inc.). Sequencing output from the NovaSeq platform was converted to fastq format and demultiplexed using Illumina Bcl2Fastq 2.20.0.

#### 2.4.3 Taxonomic Characterization

Quality control of the demultiplexed sequencing reads was verified by FastQC 0.11.8 [11]. Adapters were trimmed using TrimGalore 0.6.2 [12]. The resulting paired-end reads were classified with Kraken 2.1.2 [13] and Bracken 2.5 [14] and all reads identified as host were eliminated. Reads unclassified by Kraken2 were submitted to Spades 3.14.1 for meta-assembly [15]. Contigs in the meta-assembly of length greater than 500 were submitted to BLAST 2.12.10 [16] for potential taxa identification. Reads were mapped back to an index of the contigs via Bowtie2 2.4.1 [17] for quantification. Contigs of the meta-assembly of reads identified as unknown were submitted to DFAST 1.2.15 [18] for annotation. Annotations were submitted to MetaQUAST 5.0.2 [19] for quality assessment. Taxa identified in the meta-assemblies of the unclassified reads were evaluated for suitability of inclusion in the Kraken2 database. Genomic references for suitable taxa were added from GenBank based on whether they were assembled, and at least specified a genus. This process continued for three iterations, at which point no suitable taxa were recovered. All reads identified as not being derived from host, including those that remained unclassified were submitted to Spades for meta-assembly. Contigs of these meta-assemblies were submitted to BLAST and Kraken2 for potential taxa identification and reads were mapped using Bowtie2 to the contigs for quantification. Annotation and quality assessment on the reads classified as non-host was performed using DFAST and MetaQUAST respectively. Potential metabolic pathways were characterized using Humann3 [20, 21]. Raw counts from the Bracken pipeline and pathway abundance were normalized using the formula:

$$\log_{10}\left(\frac{RC}{n}\times \frac{\sum x}{N}+1\right)$$

where RC represents the individual sample OTU counts, n is the number of sequences in a sample, the sum of x is the total number of counts in the table and N is the total number of samples. To remove potentially spurious low abundance taxa, we removed all taxa or pathways with a relative abundance below 0.000168% at the species level, based on histogram distribution (data not shown) and corresponding to a mean relative abundance of 100 reads or less.

#### 2.4.4 Antimicrobial Resistance (AMR) Gene Characterization

Paired-end reads identified as non-host were joined with VSEARCH 1.17.1 [22] and queried against The Comprehensive Antibiotic Resistance Database (CARD) [23]. Non-host contigs assembled by Spades underwent antibiotic resistance gene (ARG) calling with CARD’s Resistance Gene Identifier 5.2.1 (RGI) [23] and NCBI’s Antimicrobial Resistance Gene Finder 9.27 (AMRFinderPlus) in parallel [24]. RGI was run with default parameters which used a blast query algorithm to match genes in the CARD database to those in the contigs. RGI by default reports genes matched with 95% identity (“Strict”) up to 100% identity (“Perfect”). To assure the validity of the genes reported by RGI, AMRFinderPlus was run on the same contigs using default parameters. Genes reported from AMRFinderPlus were also restricted to genes that had EXACT (100% identity) and BLAST (>90% identity). AMR genes identified using each of these three methods were normalized using RPKM (reads per kilobase of transcript per million reads mapped). Genes with 80% or more of the samples with zero counts were filtered out.

### 2.5 Statistical Analysis

During each filtering step, *i.e.,* raw reads, deduplicated reads, trimmed reads, and cleaned reads (host DNA removed), bbmap 38.96 was used to calculate read length. Read length mean and median summaries were calculated. To visualize patient sample relative to their collection date a time-scaled scatterplot was created. Taxonomic data from Bracken and antibiotic resistance gene data from each ARG caller were gathered and parsed into count tables. Samples with insufficient DNA concentration or poor-quality sequencing metrics (<0.3 million reads) were excluded from downstream analyses to minimize bias due to incomplete or low-complexity data. To prevent distortion of longitudinal or group-level comparisons, only patients with a minimum of samples at two time points were included in statistical modeling. No data imputation was performed for missing samples.

Multidimensional Scaling (MDS) was performed with R package “vegan” (version 2.5.4) at the species level and for AMR gene counts with the “Bray-Curtis” distance in the “capscale” function, and the p-values were extracted from the mixed linear models with effect of interests. Shannon diversity was calculated using the “diversity” function from the “vegan” package, and p-values were extracted from the two- sample t-test using the base R “t.test” function, or from Wilcoxon rank-sum test using the base R “wilcox.test” function. Mixed linear models were constructed using the “lme” function from the “nlme” package. Mixed linear models include subject ID as a random variable and patient group and time as fixed variables.

Statistical analyses and visualization of data was done through R version 4.2.2, and Python version 3.11.3. Adjustment for multiple hypothesis testing was performed using FDR correction.

### 2.6 Data Availability

All sequencing from the current study have been deposited in the National Center for Biotechnology Information Sequence Read Archive under the BioProject PRJNA1306109.

## 3. Results

### 3.1 Comparison of Gut Microbiomes in Patients Randomized to Home Versus Hospital HCT Demonstrates Few Differences Between Groups

Our study cohort was constructed from longitudinally sampled stools, from patients randomized to undergoing HCT either at home or in the hospital as part of Phase II trials, described previously [5]. We identified 28 patients who met inclusion criteria (see Methods); 16 of these were patients randomized to home HCT, 12 were randomized to hospital HCT. No significant differences were observed between groups for any patient- or transplant-related variables indicating appropriate randomization (Table 1). The duration of hospitalization was similar between the two groups (p = 0.199; Figure S1).

**Figure 1:**
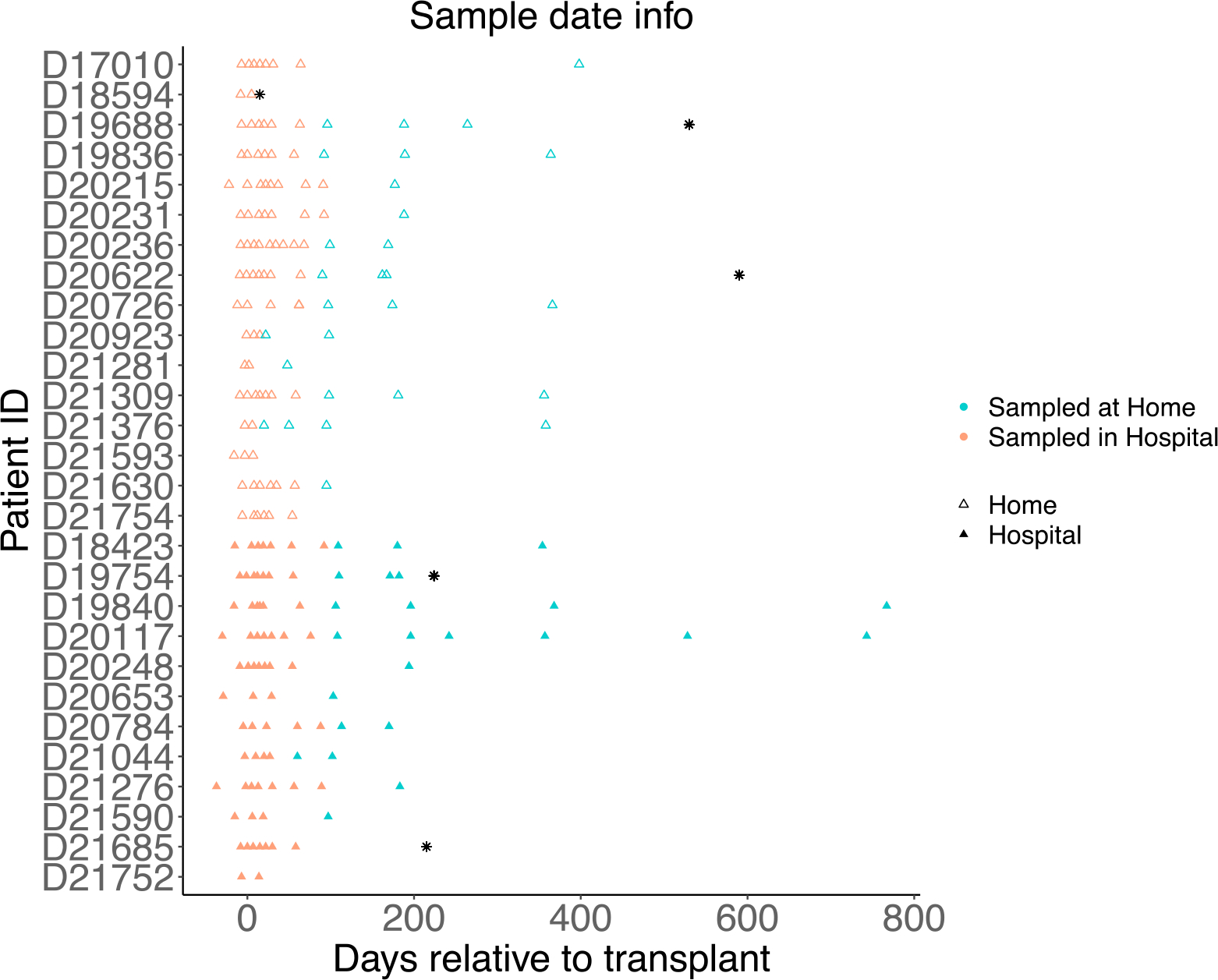
Stool samples from patients randomized to HCT at home or in the hospital are plotted for each patient relative to the day of transplantation. Samples are colored based on the location of sampling, shape based on treatment group type; black asterisks represent the days after transplant on which patients died.

Stool samples from each patient were subjected to shotgun metagenomic sequencing to a target depth of 50–60 million 150 base pair paired-end reads. 213 stools were obtained, and 203 stools from 202 samples were sequenced with 10 removed as a result of low DNA extraction concentration (sample date range −37 days prior to HCT and +767 days after HCT; Figure 1). Reads underwent both taxonomic characterization as well as characterization of antimicrobial resistance (AMR) genes via three orthogonal methods. On average, each sample was found to have a median of 3224 species (range 236–6249), while the median number of AMR genes identified varied widely between methods: 9 on average using AMR Finder (range 1–12), 54 using RGI (range 13–184), and 253 using VSEARCH (range 92–492). Sequencing depth for samples prior to quality filtration is included in Table S1.

**Figure 2:**
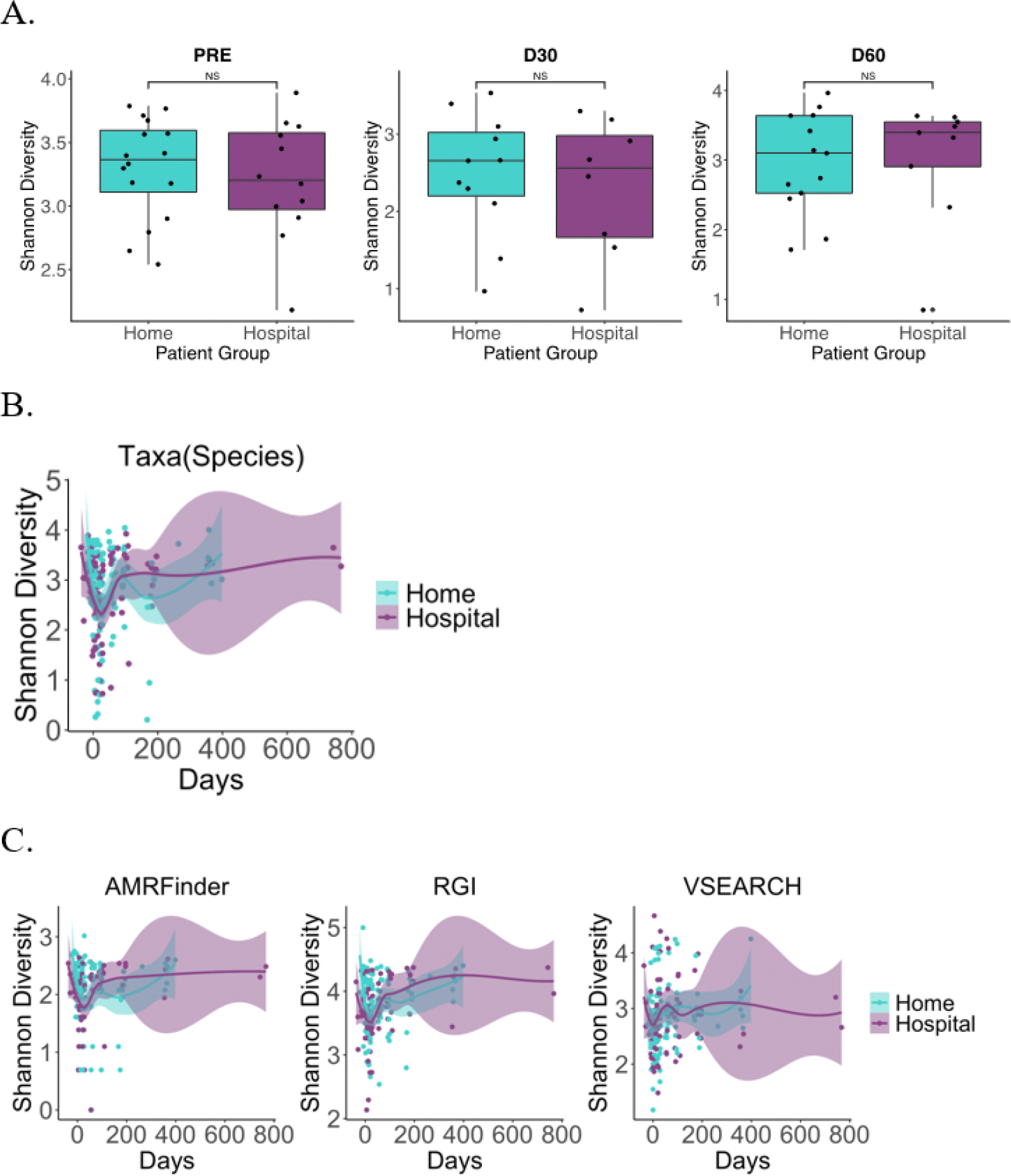
Taxonomic and AMR gene Shannon diversity did not differ between patients randomized to HCT at home or in the hospital. A) Taxonomic Shannon diversity at the species level was compared between patients undergoing home v. hospital HCT. No differences in Shannon diversity were found using the Wilcoxon rank-sum test before (p = 0.599) or after HCT at days +30 and +60 after transplant (p = 0.840 and p = 0.948, respectively). B) Shannon diversity over time at the species level is comparable between HCT groups (p = 0.519, mixed linear model). C) Shannon diversity of AMR genes characterized using three orthologous methods were not significantly different (AMRFinder, p = 0.351; RGI, p = 0.177; VSEARCH, p = 0.634, mixed linear model with home vs. hospital effect only).

To compare taxonomic and AMR gene composition between patients who had undergone HCT at home versus in the hospital, we calculated Shannon diversity (Figure 2) before HCT (pre-HCT) and at days +30 and +60 after HCT at the species level (Figure 2A), for all timepoints (Figure 2B) and for AMRFinder, RGI, and VSEARCH results (Figure 2C). Overall, we find no significant differences in taxonomic or AMR gene Shannon diversity for all patients (Figure 2) or for only allo-HCT patients (data not shown).

**Figure 3:**
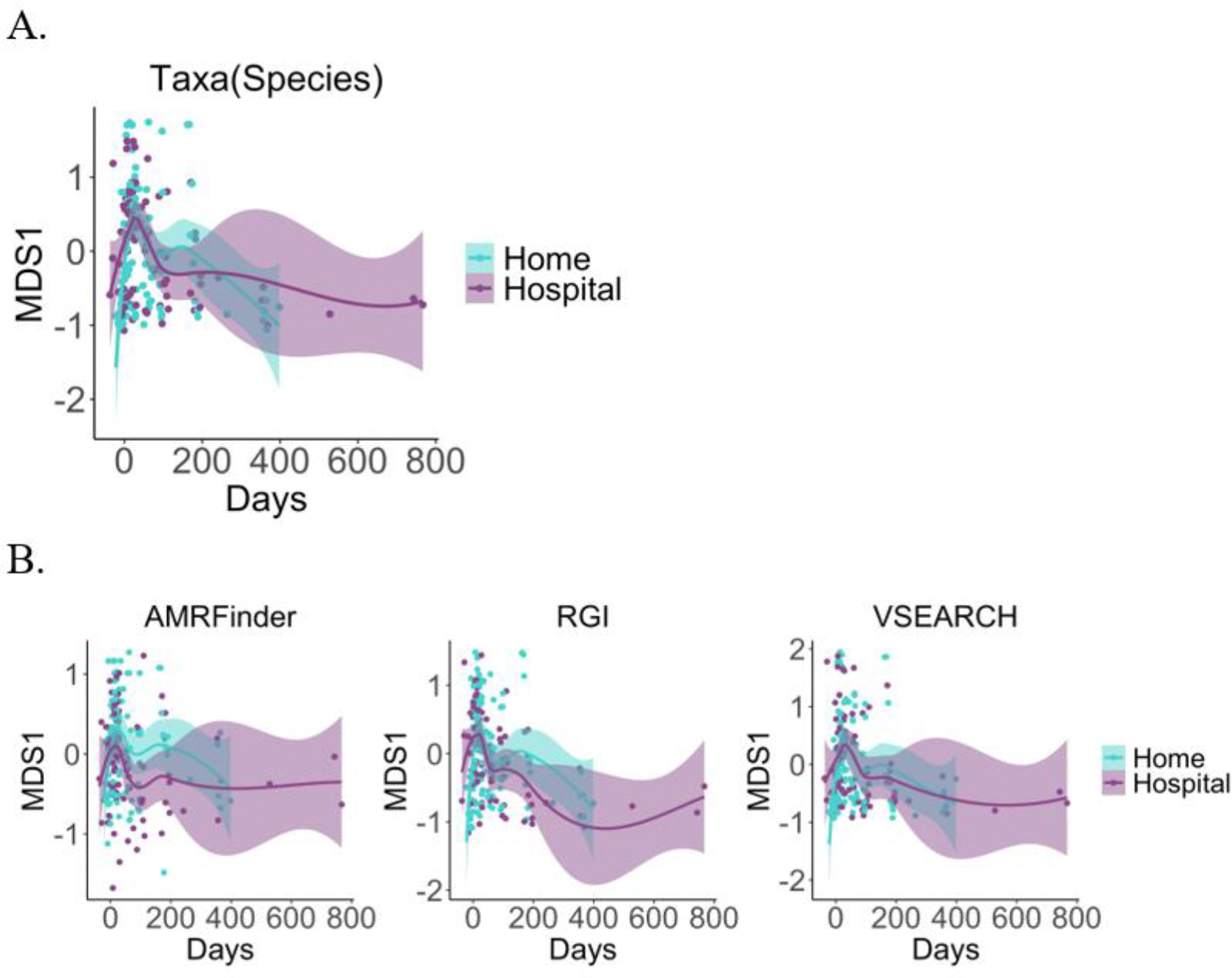
Beta-diversity of gut microbial taxonomic and AMR gene composition does not differ between home and hospital HCT. Multidimensional scaling (MDS) between patients in each group at all timepoints was performed using Bray-Curtis dissimilarity measurements and tested via mixed linear models of A) gut microbiome taxonomic composition at the species level (p = 0.482), B) and AMR genes characterized using three orthologous methods (AMRFinder, p = 0.155; RGI, p = 0.693; VSEARCH, p = 0.668).

In order to investigate taxonomic compositional, or beta-diversity, changes over time between home and hospital HCT groups, we compared Bray-Curtis dissimilarity between timepoints using multidimensional scaling (MDS); we plotted MDS1 for both taxonomic and AMR gene composition over time (Figure 3). For taxonomic composition and AMR genes, we observe no difference in beta-diversity between groups for all patients (Figure 3) or for allo-HCT patients alone (data not shown).

We constructed mixed linear models of individual taxa or AMR genes and found no statistically significant differences between groups after correction for multiple hypothesis testing (Figure S2). However, we did observe changes in the differential abundance of *Streptococcus* spp. and *Actinomyces neuslundii* over time that trended towards significance (adjusted p-values = 0.0532) with the relative abundance of these species declining or remaining stable in the home HCT group and rising in the hospital HCT group. We performed similar mixed linear models with AMR genes and metabolic pathways and did not find any significant differences between patients randomized to receive HCT at home or in the hospital (data not shown).

### 3.2 Secondary Analyses Demonstrate Differences in Gut Microbiomes Based on the Location Where Patients Spent Most of Their Time After HCT, Irrespective of Randomized Group Allocation

**Figure 4:**
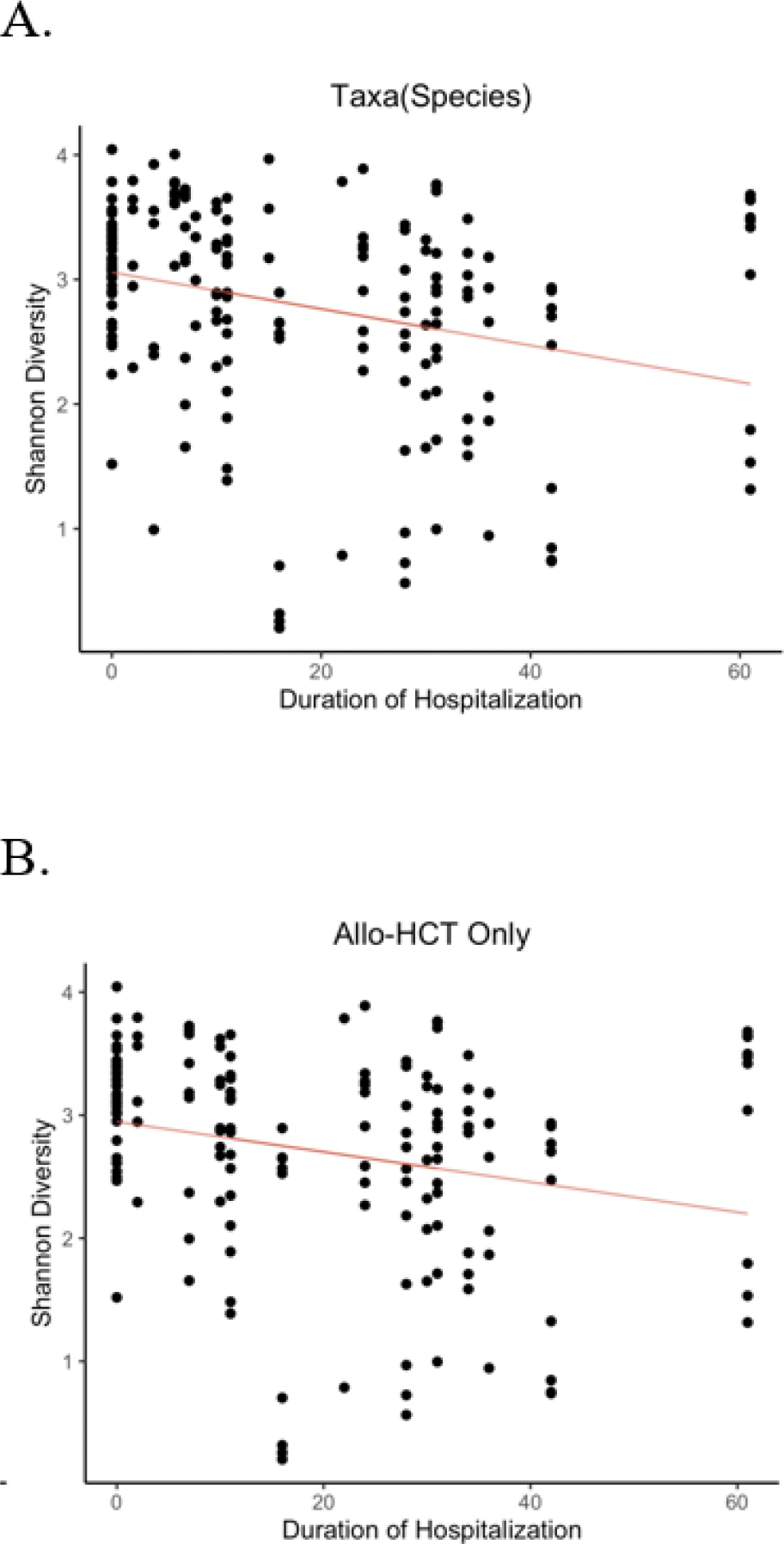
Shannon diversity was significantly lower in patients hospitalized for a longer duration. A) Taxonomic Shannon diversity at the species level was compared with hospitalization duration. Patients hospitalized for a longer duration were found to have significantly lower gut species Shannon diversity: A) when comparing all patients’ samples (p = 0.00976, mixed linear model), and B) only including those patients undergoing allo-HCT (p = 0.0439, mixed linear model).

Having observed no difference in the duration of hospitalization between patients randomized to home or hospital HCT, we sought to compare patients based on whether they spent more time in the inpatient or outpatient setting (see methods for details on inpatient v. outpatient cohort definitions; Figure S3). We found that the duration of hospitalization was significantly greater by 2 weeks for those patients allocated to receive their stem cell infusion in the inpatient setting (“inpatient”) versus those who received their infusion in the outpatient infusion clinic setting (“outpatient”) (29 days v. 5 days, p = 5.41e-05; Figure S4). We also found that in our cohort, the duration of hospitalization was inversely correlated with Shannon diversity for all patients (Figure 4A) and when considering only allo-HCT (Figure 4B). As the actual transplant location almost perfectly differentiated high versus low duration of hospitalization, in our secondary analysis we compared the gut microbiomes of these two groups (outpatient v. inpatient HCT) in order to determine the impact of hospitalization duration on gut microbial diversity.

Patient and transplant variables between the outpatient and inpatient HCT groups were for the most part comparable (Table S2). No differences were found between groups in patients’ age, race, or randomization to home v. hospital HCT groups. There were, however, significant differences in conditioning regimen and transplant type between groups with only samples from allogeneic recipients being obtained from the inpatient hospital group (p = 0.03). Although the total number of antibiotic courses were not different between groups, patients in the inpatient HCT group were significantly more likely to receive antibiotics with anaerobic activity (*e.g.,* piperacillin-tazobactam, meropenem) compared to those in the outpatient HCT group (p = 0.003).

**Figure 5:**
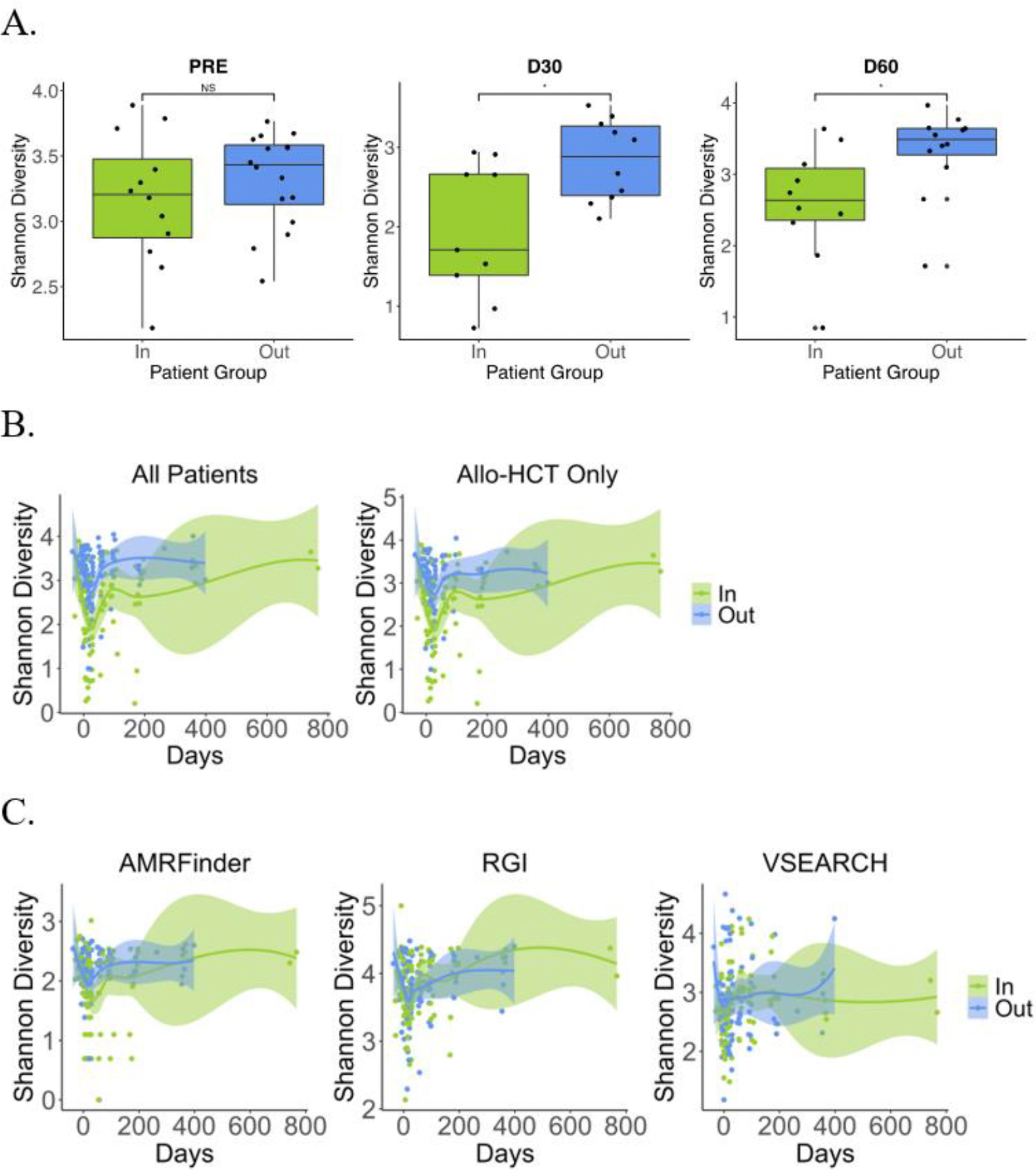
Shannon diversity was significantly higher in outpatient HCT recipients after, but not prior, to transplantation. A) Taxonomic Shannon diversity at the species level was compared between patients undergoing outpatient v. inpatient HCT. Patients in the outpatient HCT group were found to have higher gut species Shannon diversity at days +30 and +60 after transplant (p = 0.0279 and p = 0.0206 respectively) but not before HCT (p = 0.478, Wilcoxon rank-sum test). B) Shannon diversity over time at the species level is higher in the outpatient compared with the inpatient HCT group (p = 0.0000899, mixed linear model). The relative difference in taxonomic Shannon diversity between the groups remained significant even when only considering patients undergoing outpatient allo-HCT (n = 9) or inpatient allo-HCT (n = 12) (p = 0.00132, mixed linear model). C) Shannon diversity of AMR genes characterized using three orthologous methods were not significantly different (AMRFinder, p = 0.155; RGI, p = 0.645; VSEARCH, p = 0.984).

Comparing the gut microbiota between outpatient and inpatient HCT groups, we find no significant difference in Shannon diversity prior to transplantation, although outpatient HCT was significantly associated with higher diversity at both days +30 and +60 (Figure 5A; p < 0.05 by Wilcoxon test), and for all timepoints (Figure 5B; p < 0.05 by mixed linear model). In contrast, despite differential taxonomic diversity following transplant, no significant difference in AMR gene diversity was observed between groups before or after HCT (Figure 5C). As the two study groups were imbalanced in transplant type, we investigated differences in Shannon diversity throughout allo-HCT and found that Shannon diversity remained significantly higher in the outpatient allo-HCT group (Figure 5B).

**Figure 6:**
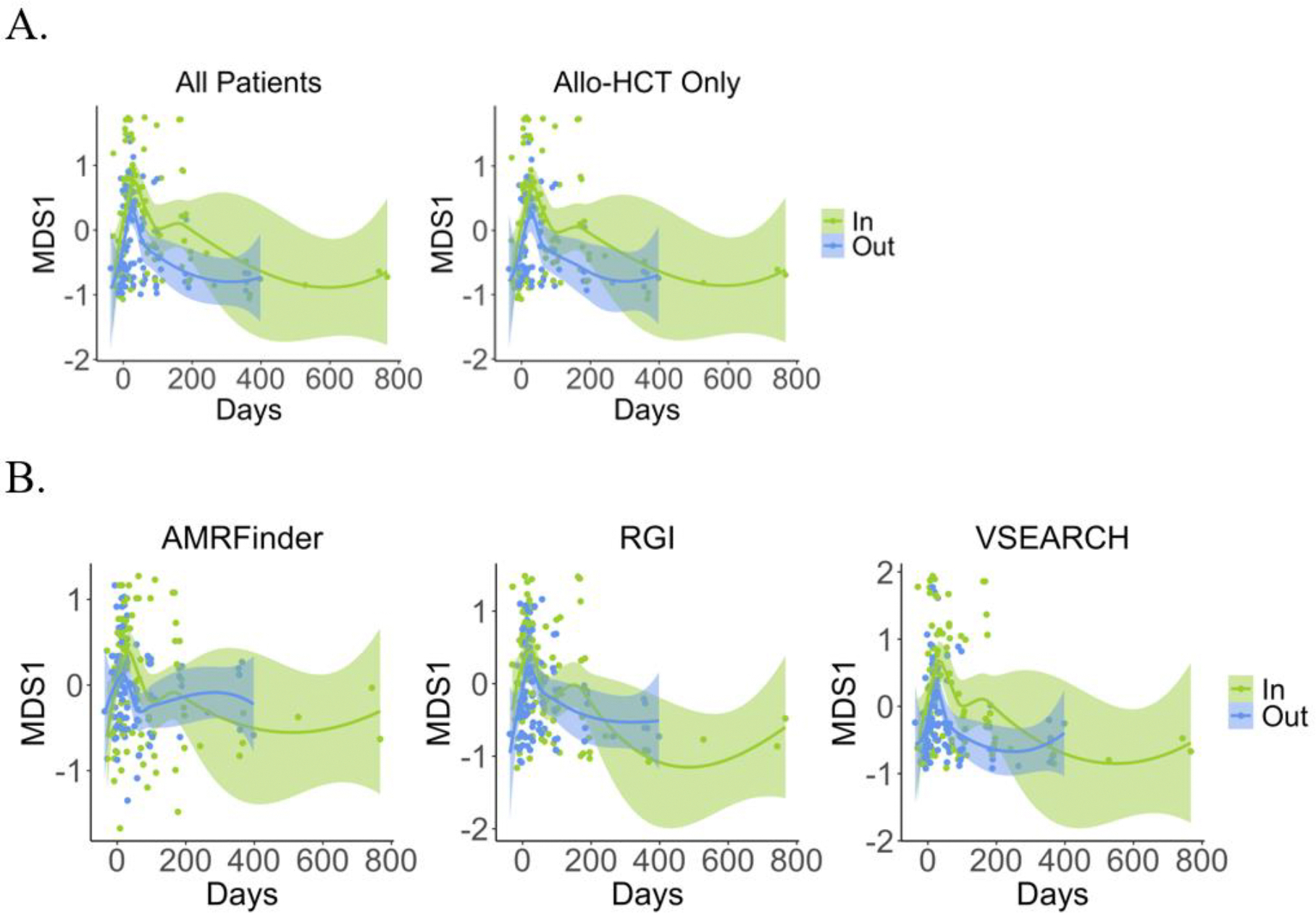
Beta-diversity of gut microbial taxonomic and AMR gene composition differs after, but not before, transplant between inpatient and outpatient HCT groups. Multidimensional scaling (MDS) between patients in each group at all timepoints was performed using Bray-Curtis dissimilarity measurements and testing via mixed linear models of A) gut microbiome taxonomic composition at the species level (p = 0.00661), when considering allo-HCT patient only (p = 0.0318), and B) AMR genes characterized using three orthologous methods (AMRFinder, p = 0.405; RGI, p = 0.110; VSEARCH, p = 0.0173).

In terms of β-diversity, for both taxonomic composition and AMR genes, we observe little difference between groups pre-HCT but more separation between inpatient and outpatient HCT at after transplant (p = 0.00661, mixed linear model) (Figure 6). Comparing differential taxonomic composition between groups over time using MDS1, β-diversity was significantly higher in outpatient HCT recipients (Figure 6A), although these same differences were not observed consistently for AMR genes (Figure 6B).

In mixed linear models, we did not find any significant differential abundance in individual taxa or AMR genes between groups over time (data not shown). These results suggest a modest effect size in beta-diversity for the relationship between transplant location and taxonomic composition, but not enough of an effect size that could be observed with individual taxa alone.

## 4. Discussion

Recent years have seen an increasing demand for home-based healthcare, recognizing the impact that prolonged hospitalization can have on patients’ quality of life. Observational studies of home versus hospital HCT have demonstrated that home-based HCT proves to be a safe alternative to inpatient treatment with benefits for patients’ overall wellbeing, however the impact that the shift from hospital to home environment and the effect of the built environment on the gut microbiome has not yet been established. In this first of its kind investigation, we demonstrate in patients randomized to home v. hospital HCT, that there were no significant differences in Shannon diversity or gut microbial taxonomic composition before or after transplant. Despite expected differences between groups, patients were hospitalized for a similar duration in those randomized to home or hospital HCT which may account for microbiome similarity. In contrast, patients in the inpatient vs. outpatient groups had significant differences in overall duration of hospitalization, and inpatient HCT recipients were found to have a significantly lower Shannon diversity and differential taxonomic composition after, but not before, transplant compared to outpatient HCT recipients. While hospitalization duration in many patient cohorts is heavily biased by indication and illness severity, HCT recipients are unique in being most commonly hospitalized until engraftment (*i.e.* an absolute neutrophil count >500 cells/mm^3^) with duration in the hospital being determined often by factors related to immune reconstitution. Although inpatient and outpatient HCT groups were comparable, to address any potential confounding, we compared only allogeneic HCT patients and found similar results, many of which were statistically significant despite the smaller group size; this rules out transplant type as a primary driver of our results. Notably, inpatient HCT recipients were more likely to have received anaerobic antibiotics compared to their outpatient HCT counterparts. Multiple prior studies have demonstrated a significant impact of anaerobically active antibiotics on the gut microbiome and worse clinical outcomes after HCT [25, 26]. Other factors such as differential dietary intake at home versus in the hospital may also be implicated in alterations to gut microbial diversity and warrant further future investigation.

Importantly, differences in diversity and taxonomic composition based on actual transplant location and duration of hospitalization, did not translate into differences in AMR gene diversity. Our analyses benefitted from an orthogonal approach to characterizing AMR genes in order to demonstrate consistency between methods, as there are no standard methods to characterize the resistome. Little is known about how the resistome in HCT recipients changes over time despite broad awareness that the gut microbiome is the source for a majority of bloodstream infections with multidrug-resistant organisms. This is the first study to investigate the impact of hospitalization on AMR gene acquisition in this population, with the resistome demonstrating no clear relationship with duration in the hospital. While this is unexpected, prior studies have shown only minimal acquisition of new AMR genes during hospitalization and only after anaerobically active broad-spectrum antibiotics were administered [27]. One possible explanation is the persistence of a core set of resistance genes within the chromosome, not carried on plasmids and therefore less subject to horizontal transfer. This background of intrinsic AMR genes is likely much less sensitive to external pressures such as hospitalization duration.

## 5. Summary

This study provides the first prospective, randomized evidence examining how transplant location—home versus hospital—influences the gut microbiome in patients undergoing HCT. Our results challenge the prevailing assumption that home HCT inherently preserves microbial diversity by revealing that randomization to care location did not significantly alter gut taxonomic or resistome profiles, likely due to equivalent durations of hospitalization in both groups. Our secondary analyses uncovered a striking association between reduced hospitalization and increased gut microbial diversity, suggesting that it is not the home environment per se, but rather the avoidance of prolonged inpatient exposure, that confers a protective effect on the gut microbiome. This finding underscores that the duration and setting of post-transplant recovery are modifiable exposures with microbiome-level consequences, potentially influencing clinical outcomes. Furthermore, this is the first study to examine the resistome in HCT recipients in relation to transplant setting and hospitalization duration, revealing no clear differences in AMR gene diversity. These findings highlight that while taxonomic diversity may fluctuate with environmental and antibiotic exposures, the gut resistome may be more stable or influenced by different factors.

Together, our data lay the groundwork for future microbiome-targeted studies aimed at reducing post-transplant complications, including larger studies that compare home versus hospital HCT and longer versus shorter durations of hospitalization. Future strategies to mitigate microbial injury during HCT should also prioritize microbiome-sparing approaches, including an emphasis on more targeted-spectrum antibiotics that minimize damage, and expansion of microbiome therapeutics under current development such as prebiotics (NCT05135351), fecal microbiota transplantation (NCT06026371), and rationally-designed microbial consortia [28].

## Supplementary Material

Supplemental figures and tables 1. Figure S1: Similar duration of hospitalization observed for patients randomized to HCT in the hospital compared to the home. We compared the duration of intensive daily transplant care using the Wilcoxon rank-sum test between patients randomized to HCT at home or in the hospital and found no significant difference between groups (p = 0.199).
2. Figure S2: Mixed linear models demonstrate no individual taxa that differ significantly between patients randomized to home or hospital HCT. Top eight most significant taxa at the species level (p-values from mixed linear models with home vs. hospital effect only).
3. Figure S3: Stool samples from patients undergoing inpatient versus oputpatient HCT are plotted for each patient relative to the day of transplantation. Samples are colored based on the location of sampling, shaped based on treatment group type; black asterisks represent the days after transplant on which patients died.
4. Figure S4: Total number of hospital days observed for patients in the inpatient HCT group is significantly higher compared to the outpatient HCT group. We compared the duration of intensive daily transplant care using the Wilcoxon rank-sum test between patients assigned to HCT in the inpatient or outpatient settings by patient (p = 5.41e-05).
5. Table S1: Pre-processed sequenced reads from stool samples, summarized per patient.
6. Table S2: Demographics and transplant characteristics of patients included, comparing patients undergoing outpatient and inpatient HCT.

## Figures and Tables

**Table 1:** Demographics and transplant characteristics of patients included, comparing patients randomized to home and hospital HCT.

		Home (n = 16)	Hospital (n = 12)	p-value

Age (median [IQR])		54.50 [35.75, 68.00]	57.50 [50.25, 61.75]	0.91
Sex (female)		6 (37.5)	4 (33.3)	1.00
Race	Asian descent	0 (0.0)	1 (8.3)	0.53
	African descent	2 (12.5)	1 (8.3)	
	European descent	13 (81.2)	10 (83.3)	
	Other	1 (6.2)	0 (0.0)	
Ethnicity^1^	Hispanic	1 (6.2)	0 (0.0)	0.35
	Non-Hispanic	15 (93.8)	11 (91.7)	
Underlying malignancy	ALL/AML	8 (50.0)	5 (41.7)	0.86
	NHL/HL	2 (12.5)	2 (16.7)	
	MM	2 (12.5)	2 (16.7)	
	MDS/MPN	3 (18.8)	3 (25.0)	
	Other	1 (6.3)	0 (0.0)	
Transplant type	Allogeneic	12 (75.0)	9 (75.0)	1.00
	Autologous	4 (25.0)	3 (25.0)	
Conditioning regimen	Myeloablative-allogeneic	7 (43.8)	7 (58.3)	0.82
	Myeloablative-autologous	4 (25.0)	3 (25.0)	
	Non-myeloablative	3 (18.8)	1 (8.3)	
	Reduced intensity	2 (12.5)	1 (8.3)	
Donor type	Matched related	5 (31.2)	4 (33.3)	0.99
	Matched unrelated	7 (43.8)	5 (41.7)	
	Autologous	4 (25.0)	3 (25.0)	
HCT-CI (median [IQR])		3.00 [1.50, 4.25]	3.00 [1.75, 3.00]	0.85
Acute GVHD		2 (16.7)	4 (44.4)	0.33
Location of infusion	Inpatient	6 (37.5)	6 (50.0)	0.78
	Outpatient	10 (62.5)	6 (50.0)	
Antibiotic prophylaxis	Fluoroquinolone	16 (100.0)	10 (83.3)	0.34
	Trimethoprim-sulfamethoxazole	13 (81.2)	10 (83.3)	1.00
Antibiotic treatment	Broad-spectrum antibiotics^2^	13 (81.2)	12 (100.0)	0.33
	Antibiotics with high anaerobic activity^3^	6 (37.5)	5 (41.7)	1.00
	IV Vancomycin	12 (75.0)	12 (100.0)	0.19

1 One patient in the Hospital HCT group with unknown ethnicity.

2 Includes ceftriaxone, ceftazidime, cefepime, piperacillin-tazobactam, meropenem, aztreonam.

3 Includes piperacillin-tazobactam, meropenem, metronidazole, amoxicillin-clavulanate.

Definitions: ALL, acute lymphocytic leukemia; AML, acute myeloid leukemia; NHL, Non-Hodgkin’s lymphoma; HL, Hodgkin’s lymphoma; MM, Multiple myeloma; MDS, Myelodysplastic syndrome; MPN, Myeloproliferative neoplasm.
