# Supplementary material for: Duration of Hospitalization is Associated with the Gut Microbiome in Patients Undergoing Hematopoietic Stem Cell Transplantation: Early Results from a Randomized Trial of Home Versus Hospital Transplantation
Source: OBM Transplant. Author manuscript; Available in PMC 2025 Oct 17. (PMC12530115; doi:10.21926/obm.transplant.2503255)
Supplement: Supplemental figures and tables — 1. Figure S1: Similar duration of hospitalization observed for patients randomized to HCT in the hospital compared to the home. We compared the duration of intensive daily transplant care using the Wilcoxon rank-sum test between patients randomized to HCT at home or in the hospital and found no significant difference between groups (p = 0.199). 2. Figure S2: Mixed linear models demonstrate no individual taxa that differ significantly between patients randomized to home or hospital HCT. Top eight most significant taxa at the species level (p-values from mixed linear models with home vs. hospital effect only). 3. Figure S3: Stool samples from patients undergoing inpatient versus oputpatient HCT are plotted for each patient relative to the day of transplantation. Samples are colored based on the location of sampling, shaped based on treatment group type; black asterisks represent the days after transplant on which patients died. 4. Figure S4: Total number of hospital days observed for patients in the inpatient HCT group is significantly higher compared to the outpatient HCT group. We compared the duration of intensive daily transplant care using the Wilcoxon rank-sum test between patients assigned to HCT in the inpatient or outpatient settings by patient (p = 5.41e-05). 5. Table S1: Pre-processed sequenced reads from stool samples, summarized per patient. 6. Table S2: Demographics and transplant characteristics of patients included, comparing patients undergoing outpatient and inpatient HCT. [file NIHMS2114751-supplement-Supplemental_figures_and_tables.pdf]

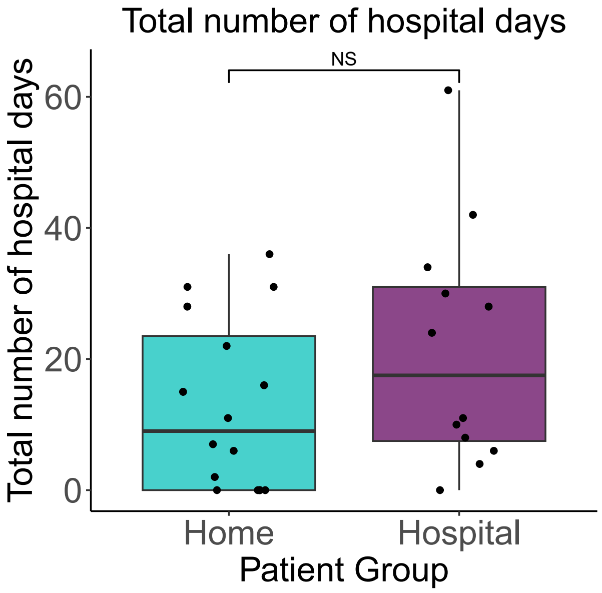

Figure S1 Similar duration of hospitalization observed for patients randomized to HCT in the hospital compared to the home. We compared the duration of intensive daily transplant care using the Wilcoxon rank-sum test between patients randomized to HCT at home or in the hospital and found no significant difference between groups (p = 0.199).

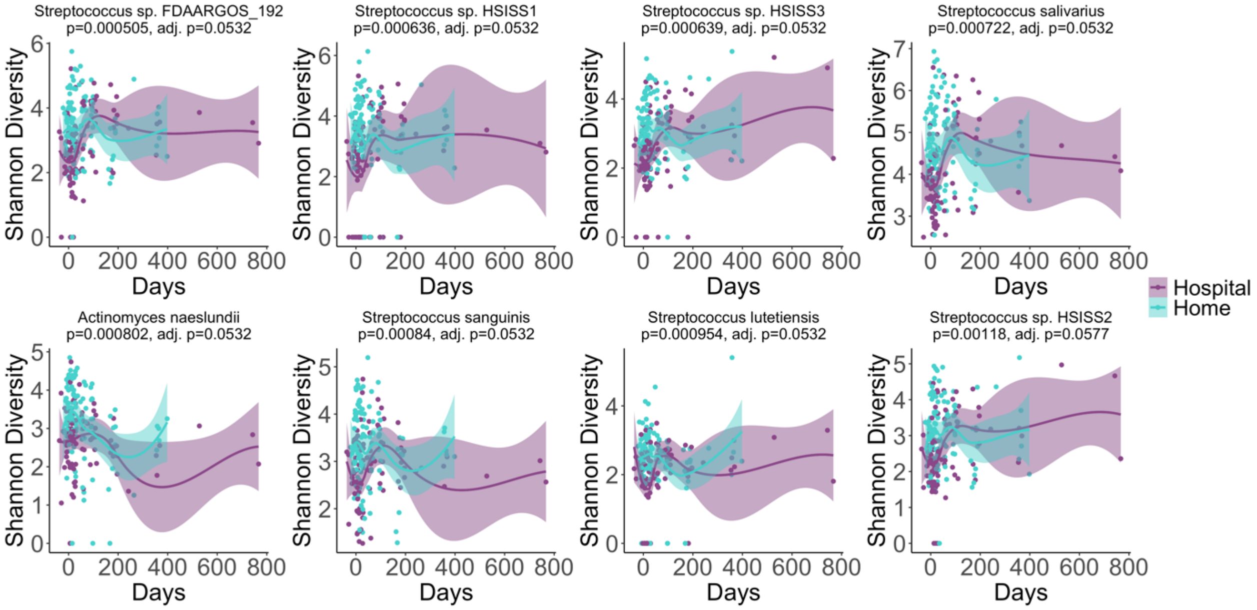

Figure S2 Mixed linear models demonstrate no individual taxa that differ significantly between patients randomized to home or hospital HCT. Top eight most significant taxa at the species level (p-values from mixed linear models with home vs. hospital effect only).

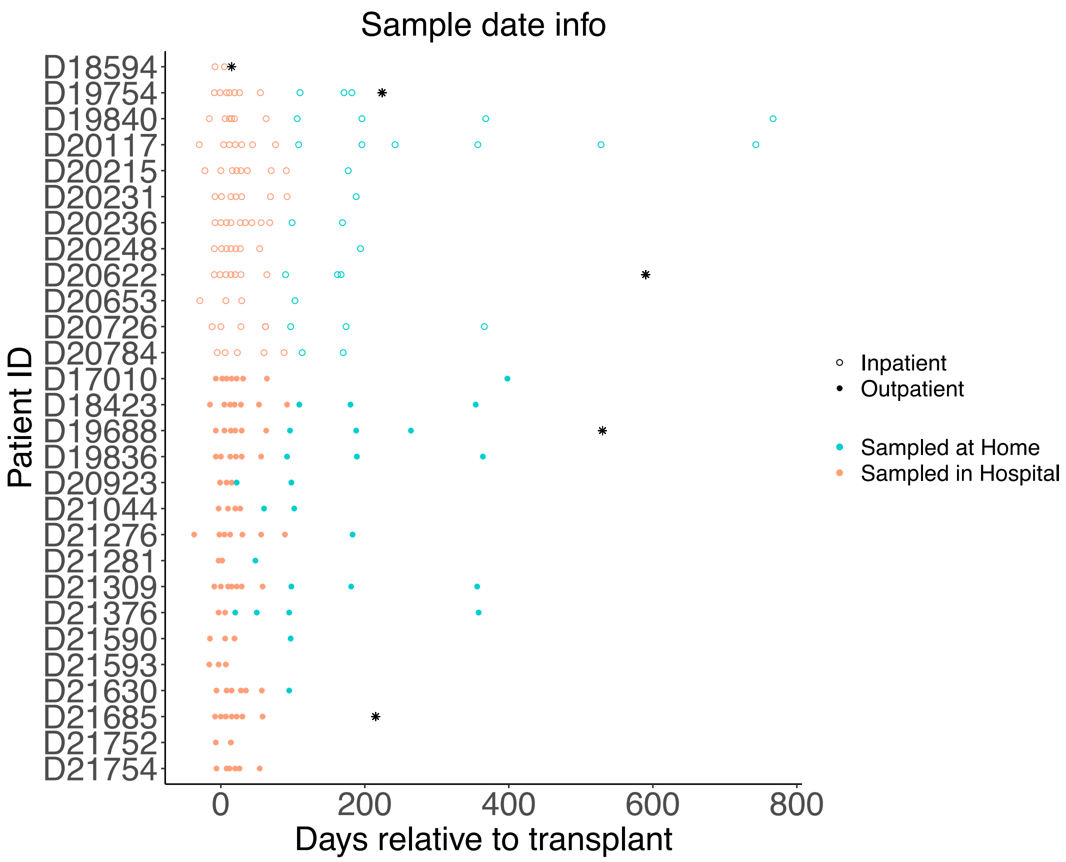

Figure S3 Stool samples from patients undergoing inpatient versus oputpatient HCT are plotted for each patient relative to the day of transplantation. Samples are colored based on the location of sampling, shaped based on treatment group type; black asterisks represent the days after transplant on which patients died.

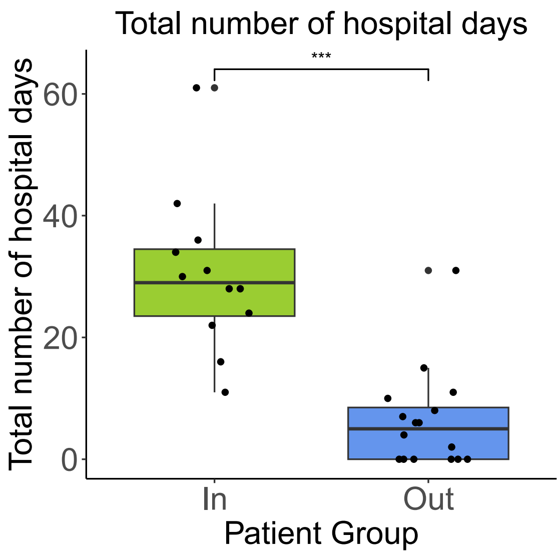

Figure S4 Total number of hospital days observed for patients in the inpatient HCT group is significantly higher compared to the outpatient HCT group. We compared the duration of intensive daily transplant care using the Wilcoxon rank-sum test between patients assigned to HCT in the inpatient or outpatient settings by patient ($p = 5.41e{-}05$).

Table S1 Pre-processed sequenced reads from stool samples, summarized per patient.

Patient	Number of stools	Median read count	Lower Quartile	Upper Quartile	Home HCT	Outpatient HCT
D17010	8	63641298	59190302	70778088	Yes	Yes
D18423	10	60114742	48695433	66687636	No	Yes
D18594	2	63979607	58763682	69195533	Yes	No
D19688	9	65913108	63208680	71647210	Yes	Yes
D19754	10	58256599	51695929	69058570	No	No
D19836	9	64961930	59699736	65907098	Yes	Yes
D19840	10	65902016	58847253	73879245	No	No
D20117	13	72550732	56392238	76911434	No	No
D20215	9	61059838	55157922	70186590	Yes	No
D20231	8	60164017	58531106	63383528	Yes	No
D20236	11	67827998	53542874	74723125	Yes	No
D20248	8	70633281	66473931	72972006	No	No
D20622	10	68060656	63695948	67599122	Yes	No
D20653	4	60003914	52255023	57462772	No	No
D20726	8	57732599	53852301	67301276	Yes	No
D20784	7	65299692	62754983	72286570	No	No
D20923	5	67037162	61424842	69349338	Yes	Yes
D21044	6	62349530	49348455	73976697	No	Yes
D21276	8	63267538	52536507	71018611	No	Yes
D21281	3	65717240	62557028	66659232	Yes	Yes
D21309	10	60222643	47013591	64653492	Yes	Yes
D21376	6	63203735	55665301	60702230	Yes	Yes
D21590	4	61454039	60333469	63215233	No	Yes
D21593	3	64198660	62249311	65877142	Yes	Yes
D21630	7	61045826	50400880	63381246	Yes	Yes
D21685	7	69097984	60288307	71917923	No	Yes

D21752	2	46895854	46845740	46795626	No	Yes
D21754	6	67540916	59453663	70957588	Yes	Yes

Table S2 Demographics and transplant characteristics of patients included, comparing patients undergoing outpatient and inpatient HCT.

		**Inpatient (n = 12)**	**Outpatient (n = 16)**	**p-value**
Age (median [IQR])		52.00 [41.00, 60.25]	60.50 [43.00, 67.25]	0.46
Sex (female)		6 (50.0)	4 (25.0)	0.33
Race	Asian descent	1 (8.3)	0 (0.0)	0.41
	African descent	2 (16.7)	1 (6.2)	
	European descent	9 (75.0)	14 (87.5)	
	Other	0 (0.0)	1 (6.2)	
Ethnicity[1]	Hispanic	1 (8.3)	0 (0.0)	0.35
	Non-Hispanic	11 (91.7)	15 (93.8)	
Underlying malignancy	ALL/AML	8 (66.7)	5 (31.3)	**0.02**
	NHL/HL	0 (0)	4 (25.0)	
	MM	0 (0)	4 (25.0)	
	MDS/MPN	4 (33.3)	2 (12.5)	
	Other	0 (0)	1 (6.3)	
Transplant type	Allogeneic	12 (100.0)	9 (56.2)	**0.03**
	Autologous	0 (0.0)	7 (43.8)	
Conditioning regimen	Myeloablative-allogeniec	11 (91.7)	3 (18.8)	
	Myeloablative-autologous	0 (0.0)	7 (43.8)	**0.002**
	Non-myeloablative	1 (8.3)	3 (18.8)	
	Reduced intensity	0 (0.0)	3 (18.8)	
Donor type	Matched related	4 (33.3)	5 (31.2)	**0.02**
	Matched unrelated	8 (66.7)	4 (25.0)	
	Autologous	0 (0)	8 (47.1)	
HCT-CI (median [IQR])		2.50 [0.75, 3.00]	3.00 [2.00, 4.25]	0.24

Acute GVHD		5 (41.7)	1 (11.1)	0.18
Randomized to home HCT		6 (50.0)	10 (62.5)	0.78
Antibiotic prophylaxis	Fluoroquinolone	12 (100.0)	14 (87.5)	0.596
	Trimethoprim-sulfamethoxazole	11 (91.7)	12 (75.0)	0.522
Antibiotic treatment	Broad-spectrum antibiotics[2]	12 (100.0)	13 (81.2)	0.332
	Antibiotics with high anaerobic activity[3]	9 (75.0)	2 (12.5)	**0.003**
	IV Vancomycin	12 (100.0)	12 (75.0)	0.185

[1]One patient in the Home HCT group with unknown ethnicity.

[2]Includes ceftriaxone, ceftazidime, cefepime, piperacillin-tazobactam, meropenem, aztreonam.

[3]Includes piperacillin-tazobactam, meropenem, metronidazole, amoxicillin-clavulanate.

Definitions: ALL, acute lymphocytic leukemia; AML, acute myeloid leukemia; NHL; MM; MDS; MPN.
